# Urethrocutaneous fistula and subcutaneous abscess in the inguinal region with bacteremia caused by *Bilophila wadsworthia* in a Japanese patient: A case report

**DOI:** 10.1016/j.idcr.2025.e02147

**Published:** 2025-01-07

**Authors:** Kento Furuya, Naoya Itoh

**Affiliations:** a Division of Infection Control and Prevention, Shizuoka General Hospital, Shizuoka, Japan; b Department of Internal Medicine, Izu Redcross Hospital, Shizuoka, Japan; c Department of Infectious Diseases, Graduate School of Medical Sciences, Nagoya City University, Aichi, Japan; d Department of Clinical Infectious Diseases, Graduate School of Medical Sciences, Nagoya City University, Aichi, Japan; e Department of Infectious Diseases, Nagoya City University East Medical Center, Aichi, Japan; f Division of Infectious Diseases, Aichi Cancer Center Hospital, Aichi, Japan

**Keywords:** *Bilophila wadsworthia*, Piperacillin-tazobactam, Subcutaneous abscess, Urethrocutaneous fistula

## Abstract

*Bilophila wadsworthia* is an anaerobic, gram-negative bacillus commonly associated with acute appendicitis. However, bacteremia is exceedingly rare. Herein, we report a case of *B. wadsworthia* bacteremia associated with a urethrocutaneous fistula and a subcutaneous abscess in the left inguinal region. A 75-year-old man was referred to our hospital due to persistent fever despite piperacillin treatment. The patient was diagnosed with a urethrocutaneous fistula and a subcutaneous abscess in the left inguinal region. *B. wadsworthia* was isolated from his blood culture and identified by matrix-assisted laser desorption ionization-time of flight mass spectrometry. Subsequently, the patient underwent a four-week course of piperacillin-tazobactam therapy. Since a susceptibility breakpoint has not been established for *B. wadsworthia*, standardized treatment guidelines are currently unavailable. This case represents the first successful treatment of *B. wadsworthia* bacteremia with piperacillin-tazobactam, suggesting it may be an effective therapeutic option for infections caused by *B. wadsworthia*.

## Introduction

*Bilophila wadsworthia* is an anaerobic, gram-negative rod commonly found in the human intestinal tract [1]. It is frequently associated with acute appendicitis [1]. However, only nine cases of *B. wadsworthia* bacteremia have been reported, all of which involved intra-abdominal sources of infection, including acute appendicitis [2], [3], [4], [5], [6], [7], [8]. Moreover, the optimal treatment for *B. wadsworthia* bacteremia remains unclear. We present a case of *B. wadsworthia* infection identified in the blood culture from a patient with a urethrocutaneous fistula and a subcutaneous abscess. *B. wadsworthia* was identified using matrix-assisted laser desorption ionization-time of flight mass spectrometry, and the patient was successfully treated with piperacillin-tazobactam.

## Case Report

**Fig. 1:**
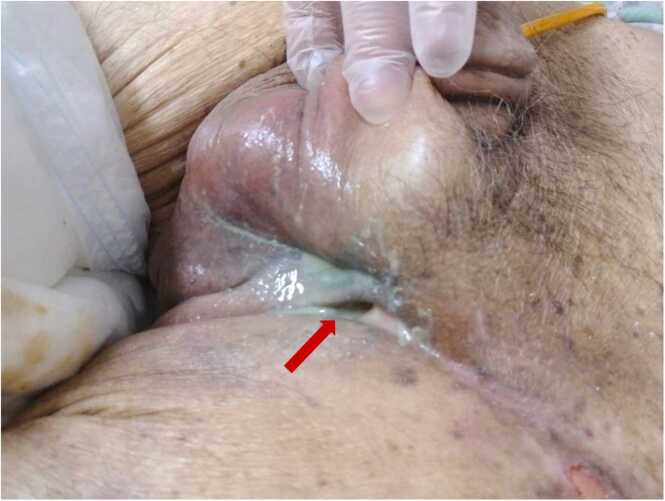
Urine and pus are draining from a urethrocutaneous fistula in the patient's left inguinal region (red arrow).

**Fig. 2:**
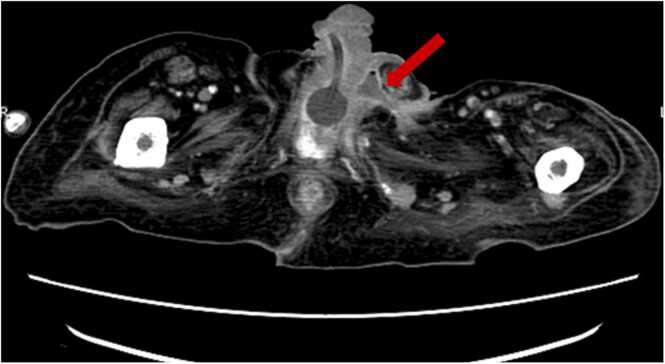
Contrast computed tomography image of the abdominopelvic region showing a subcutaneous abscess connected to a urethrodermal fistula (red arrow).

A 75-year-old man with a medical history of lower limb paralysis due to cervical spondylosis, a neurogenic bladder, and an enlarged prostate, who was previously hospitalized at another facility with an indwelling urethral catheter, developed a fever. Despite the administration of piperacillin, his fever persisted, and he gradually developed swelling and a burning sensation in the left inguinal area and scrotum. Subsequently, the patient was transferred to our hospital due to the formation of a urethrocutaneous fistula in the left inguinal region. His body temperature was 37.6°C, blood pressure was 87/34 mmHg, pulse rate was 83/min, respiratory rate was 12/min, and oxygen saturation was 95 % in room air. Redness and a burning sensation were observed from the left inguinal area to the scrotum. An ulcer had developed in part of the left inguinal region, with drainage of pus and urine noted (Fig. 1). Contrast-enhanced abdominopelvic computed tomography (CT) revealed an inflated urethral balloon catheter within the urethra and an abscess in the left inguinal region (Fig. 2).

**Fig. 3:**
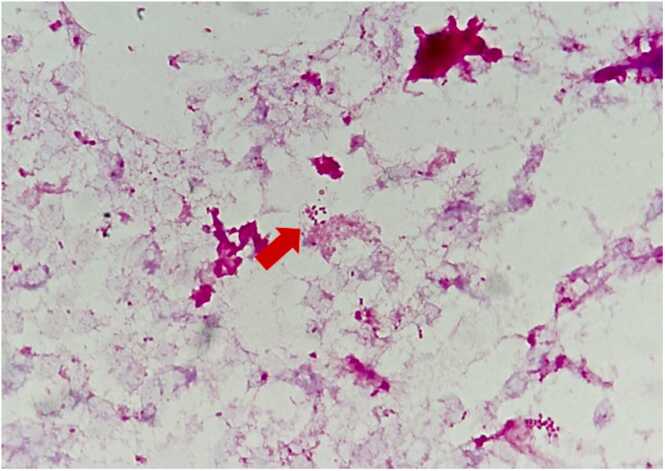
Blood culture demonstrating small gram-negative bacilli (red arrow, Gram staining, 1000 × magnification).

We administered piperacillin-tazobactam (18 g/day) and vancomycin, along with daily wound care. Blood, urine, and pus cultures were collected. The BACTEC FX (Becton Dickinson Co., Ltd., Tokyo, Japan) system signaled positive growth after 98.1 hours of incubation. Gram-negative bacilli were detected in an anaerobic blood culture bottle (Fig. 3). The positive bottle was subcultured on the sheep blood agar plate (Becton Dickinson Co., Ltd., Tokyo, Japan) and Brucella HK (RS) agar plate (Kyokuto Pharmaceutical Co., Ltd., Tokyo, Japan). *B. wadsworthia* was identified by matrix-assisted laser desorption/ionization time-of-flight mass spectrometry (MALDI Biotyper, Bruker Japan, Ltd., Kanagawa, Japan), with a score value of 2.2. According to the methodology recommended by the Clinical and Laboratory Standards Institute, document M100-S25 (2015) minimal inhibitory concentration (MIC) breakpoints for the *Bacteroides fragilis* group, the MIC of antimicrobial agents determined by broth microdilution using BD Phoenix Automated Microbiology System (Becton Dickinson Co., Ltd., Tokyo, Japan) for *B. wadsworthia* isolates from blood cultures are shown in Table 1. *Escherichia coli*, *Enterococcus faecalis*, *Pseudomonas aeruginosa*, and *Klebsiella oxytoca* were identified in his urine culture. The culture of pus from the left inguinal region showed *E. coli, Providencia stuartii, Corynebacterium striatum, Arcanobacterium haemolyticum*, and *Actinomyces turicensis*. Notably, *B. wadsworthia* was not detected in the urine and pus cultures.

**Table 1:** Antimicrobial susceptibility of isolated *Bilophila wadsworthia*.

Antimicrobial agent	MIC (µg/mL)
Penicillin G	> 1
Ampicillin	> 1
Ampicillin-sulbactam	16
Piperacillin-tazobactam	32
Cefmetazole	32
Ceftriaxone	8
Meropenem	4
Moxifloxacin	≤ 0.25
Clindamycin	≤ 0.12
Metronidazole	≤ 2

MIC: minimum inhibitory concentration

On the second day of admission (day 2), we replaced the urethral catheter and performed cystography, which revealed vesicourethral reflux. The patient was diagnosed with *B. wadsworthia* bacteremia associated with a urethrocutaneous fistula and subcutaneous abscess caused by urethral balloon misplacement. On day 3, vancomycin was discontinued. Hyperbaric oxygen therapy initiated on day 6 and continued for two weeks. We discontinued piperacillin-tazobactam on day 29 after pus drainage from the subcutaneous abscess in the inguinal region had resolved, and there was no further deterioration of the urethrocutaneous fistula. No recurrence of infection was observed after the completion of antibiotic therapy, and the patient was discharged on day 39. We did not perform lower gastrointestinal tract endoscopy because the results of two sets of fecal occult blood tests were negative.

## Discussion

We present a case of *B. wadsworthia* bacteremia with a urethrocutaneous fistula and subcutaneous abscess in the left inguinal region. There is no established consensus on the treatment of *B. wadsworthia* bacteremia; however, in this case, the patient was successfully treated with piperacillin-tazobactam.

*B. wadsworthia* is an anaerobic Gram-negative rod, typically constituting only 0.01 % of the normal human gut microbiota [1]. Among the cases of anaerobic bacteremia, the percentage of those caused by *B. wadsworthia* infection is as low as 0–0.07 % [9], [10], although *B. wadsworthia* is the third most common anaerobe detected in perforated appendicitis [1]. However, there have been only nine cases reported of *B. wadsworthia* bacteremia (Table 2) [2], [3], [4], [5], [6], [7], [8]. The reported ages of these patients ranged from 43 to 91 years, with seven being male, while the sex of the remaining two patients was not specified [2], [3], [4], [5], [6], [7], [8]. In most cases, the source of infection was intra-abdominal, except for two cases where the source remained unidentified.

**Table 2:** Summary of published cases of *Bilophila wadsworthia* bacteremia.

Case	Age	Sex	Source of infection	Other microorganisms detected in blood cultures	Treatment	Duration of antibiotic therapy	Outcome	Reference
No.1	N/A	N/A	Biliary and gastrointestinal	*Enterococcus raffinosus* *Escherichia.coli* *Klebsiella pneumoniae*	N/A	N/A	N/A	2
No.2	N/A	N/A	Biliary and gastrointestinal	Monobacteremia	N/A	N/A	N/A	2
No.3	43	Male	Liver abscess	Viridans group streptococci *Veillonella parvula*	ABPC/SBT+GM+AMB and Abscess drainage	N/A	Improved	3
No.4	62	Male	Liver abscess	*Bacteroides fragilis,* *Bacteroides uniformis,* *Clostridium cadaveris,* *Streptococcus pneumoniae*	AMPC+GM+MNZ and Abscess drainage	N/A	Improved	3
No.5	91	Male	Rectal abscess	Monobacteremia	N/A	N/A	N/A	4
No.6	70	Male	Bacterial translocation	*Bacteroides fragilis* *Eggerthella lenta* *Ruminococcus gnavus*	MEPM+MNZ	4 weeks	Improved	5
No.7	66	Male	N/A	Monobacteremia	No treatment	N/A	N/A	6
No.8	63	Male	Acute appendicitis	Monobacteremia	CEZ+MNZ and Appendectomy	3 days	Improved	7
No.9	69	Male	N/A	Monobacteremia	CTRX+CLDM	5 days	Improved	8
Present case	75	Male	Urethrocutaneous fistula Subcutaneous abscess	Monobacteremia	PIPC/TAZ and Abscess drainage	4 weeks	Improved	

ABPC/SBT; ampicillin-sulbactam, AMB; amphotericin B, AMPC; amoxicillin, CEZ; cefazolin, CLDM; clindamycin, CTRX; ceftriaxone, GM; gentamicin, MNZ; metronidazole, PIPC/TAZ; piperacillin tazobactam

In this particular case, we postulate that the urethrocutaneous fistula and subcutaneous abscess may have served as potential entry routes of *B. wadsworthia* into the bloodstream. Interestingly, *B. wadsworthia* was not detected in either the urine or pus specimens. This lack of detection may be attributed to the failure to perform anaerobic culture in both specimen examinations. Although we did not perform lower gastrointestinal endoscopy, we considered it unlikely that *B. wadsworthia* entered the bloodstream via an intraperitoneal route, based on the negative stool occult blood test result, absence of abnormal findings in the intestinal tract, including the appendix, on CT, and absence of abdominal symptoms.

This is the first reported case of urethrocutaneous fistula and subcutaneous abscess in the inguinal region with *B. wadsworthia* bacteremia successfully treated with piperacillin-tazobactam. The absence of an established breakpoint for *B. wadsworthia* results in a lack of standardized treatment guidelines [11]. Reports of resistance to clindamycin and metronidazole in *B. wadsworthia* are rare; three cases were managed with metronidazole (Cases No. 4, 6, and 8; Table 2), and one case with clindamycin (Case No. 9; Table 2) [3], [5], [7], [8]. Regarding β-lactam antibiotics, it is important to note that over 85 % of *B. wadsworthia* strains produce β-lactamase, leading to resistance against ampicillin, amoxicillin, and piperacillin [4]. Although β-lactamase production was not evaluated in our case, the detection of *B. wadsworthia* in blood cultures during piperacillin therapy suggests the possibility of resistance. This case highlights the potential efficacy of piperacillin-tazobactam in the treatment of *B. wadsworthia* bacteremia. In addition to piperacillin-tazobactam, ampicillin-sulbactam may also be effective, as demonstrated by the successful treatment of case No. 3 with this regimen [3]. In Case No. 4, amoxicillin was administered; however, its efficacy remains unclear since it was given in combination with metronidazole [3].

Initially, this case was suspected to involve a urethrocutaneous fistula stemming from an infection in the inguinal region. However, a CT scan revealed that the urethrocutaneous fistula was iatrogenic in origin, caused by an inflated urethral balloon. A urethrocutaneous fistula can result from medical interventions, infections, or trauma [12]. Patients with spinal cord injuries are particularly vulnerable to intraurethral Foley catheter balloon overinflation due to decreased sensation in the urethra, urethral sphincter spasm, and pseudourethra formation resulting from prior urethral trauma [13]. In this case, lower body paresthesia was also attributed to cervical spondylosis. When encountering a urethrocutaneous fistula, especially in instances involving lower body paresthesia, it is essential to verify the position of the urethral balloon and ensure correct inflation within the urethra.

In conclusion, we report a case of *B. wadsworthia* bacteremia in a patient with a urethrocutaneous fistula and a subcutaneous abscess in the inguinal region that was successfully treated with piperacillin-tazobactam. Although there is no consensus of the treatment for *B. wadsworthia* infection, this case shows that piperacillin-tazobactam administration can be an option.

## Ethical approval

Not applicable.

## Funding

This work was supported by the Department of Clinical Infectious Diseases, Nagoya City University Graduate School of Medical Sciences. The content is solely the responsibility of the authors and does not represent the views of the sponsor.

## CRediT authorship contribution statement

**Kento Furuya:** Writing – review & editing, Writing – original draft. **Naoya Itoh:** Writing – review & editing.

## Declaration of competing interest

The authors declare that they have no known competing financial interests or personal relationships that could have appeared to influence the work reported in this paper.

## Data Availability

All the relevant data are contained in the report.
